# Refining pancreatic ductal adenocarcinoma molecular subtype and precision therapeutics with single-nucleus RNA-seq

**DOI:** 10.1186/s12916-021-02052-y

**Published:** 2021-08-18

**Authors:** Lishu He

**Affiliations:** Department of Pharmacology & Toxicology, Medical College of Wisconsin, Milwaukee, WI USA

## Introduction

Pancreatic cancer is predicted to become the second leading cause of cancer-related deaths in the USA by 2030 with a 5-year survival rate of only 9% [1, 2]. Pancreatic ductal adenocarcinoma (PDAC) is the most common form of pancreatic malignancy and remains a treatment-refractory disease. So far, apart from surgical resection, conventional chemotherapies, radiotherapies, few therapeutic strategies, adjuvant or neoadjuvant, are available for improved PDAC patient benefit. In recent years, more research efforts have been devoted to developing targeted therapeutics via discovery of novel molecular vulnerabilities and molecular subtyping of various diseases. For PDAC, despite many attempts to refine its molecular taxonomy over the years, the current molecular subtyping still does not efficiently inform novel molecular vulnerabilities for the development of targeted therapies. Thus, fine-tuning the resolution of PDAC molecular subtyping has become a pivotal need.

Here, we discuss a manuscript from Hwang et al. which focused on the optimization of a single-nucleus RNA-sequencing (snRNA-seq) technique to understand how preoperative treatment may impact residual tumors. Moreover, we examine how this technique can further contribute to the field by identifying additional molecular vulnerabilities that can be harnessed for informative stratification during PDAC patient clinical management and targeted combinations with neoadjuvant therapies [3].

## Use of snRNA-seq for frozen archival PDAC specimen

Single-cell technologies, especially single-cell RNA-seq, have been regularly used in elucidating intertumoral tumor microenvironment (TME) and heterogeneity as well as expanding upon data from traditional bulk RNA profiling of various tumors. However, the use of scRNA-seq is not as prevalent in PDAC as in other cancers due to high intrinsic nuclease content and dense desmoplastic stroma. High-resolution transcriptional networks and any resulting patient stratification from bulk RNA analyses also tend to be obscured because of lower-quality sample collection due to the complicated PDAC TME. Hwang and colleagues propose an optimized snRNA-seq technique for better recovery of PDAC cancer cell profile without compromising the spectrum of cell states. The authors demonstrate, with frozen archival specimens not commonly considered for analysis, the potential of snRNA-seq to capture various cell types with comparable quality to that obtained from gold standard multiplex profiling *in situ*. This technique also enables processing of banked frozen tissue samples dating back at least 7 years, while bypassing some of the challenges involved in sample preparation for traditional single-cell RNA-seq, such as balancing sample viability and accurate cell type representation. Thus, the use of snRNA-seq provides an exciting avenue to further resolve PDAC molecular subtypes and gain novel insights for precision medicine approaches based on tumor reprogramming profile.

## Refined molecular taxonomy informing prognostic patient stratification

Apart from accurately capturing the malignant and non-malignant compartments of human PDAC tumors, the snRNA-seq analyses, combined with spatially resolved transcriptomics, also revealed a novel, clinically relevant molecular taxonomy with better patient stratification potential than that from the two previously identified consensus molecular subtypes, *basal-pancreatic* and *classical-pancreatic* [4]. Under the refined molecular taxonomy of PDAC proposed in the preprint, patients can be further stratified into prognostic risk groups based on malignant cell and cancer-associated fibroblast programs beyond just their conventional, consensus tumor profile.

## Multi-compartment reprogramming after neoadjuvant treatment

As a demonstration of the optimized snRNA-seq technique, Hwang et al. studied differential gene expression programs and cell types across untreated and neoadjuvant chemotherapy and radiotherapy (CRT)-treated frozen archival PDAC samples. Following CRT, PDAC tumors experienced an increase in immune filtration along with a shift in cell composition and transcriptional programming from a more classical-like profile to a more basal-like profile. Interestingly, a more immunogenic environment, despite the induced pro-resistance cell state to CRT, may render the tumor more susceptible to immunotherapies. As PDAC treatments evolve into the era of precision medicine and combination therapies, future studies are warranted to explore CRT’s potential sensitization of PDAC with CRT-induced basal-like phenotype to some immunotherapies. It would also be of interest to examine multi-compartment reprogramming following combinatorial treatments of CRT and targeted immunotherapies based on patient stratification.

## Conclusions

In summary, Hwang et al. have developed an optimized snRNA-seq technique with frozen archival specimens to further dissect the PDAC transcriptional response to CRT and refine clinically relevant molecular subtypes. The authors identify differential cell state programs in pre- and post-CRT malignant cells and demonstrate a refined molecular taxonomy of PDAC.

## Potential limitations

It should be noted, however, that the study was limited in that it did not include matched pre-and post-treatment specimens as an additional control or a sample size large enough to exclude the potential impact of the patient’s specific treatment regimen. As the frozen archival samples only included primary tumors, it would also be valuable in future studies to include metastatic tumor samples in analyses. There have also been other studies establishing PDAC patient stratification via transcriptomic signatures with cancer-associated fibroblast programs and microenvironment features taken into account ([5], for example). It would better inform the utility of snRNA-seq technique discussed here in clinical management to compare stratification patterns by those studies to the snRNA-seq-based stratification.

Nevertheless, Hwang and colleagues brought forth an impressive study supporting the increasing value of single-nucleus sequencing technologies to resolve cell state dynamics of complicated, treatment-refractory malignancies, such as PDAC, and better stratify patients for targeted therapy based on their molecular subtypes.
